# A new tool in percutaneous anterior odontoid screw fixation

**DOI:** 10.1186/s12891-020-03929-4

**Published:** 2021-01-18

**Authors:** Yan Wang, Min Li, Guanxing Cui, Jing Li, Zhiliang Guo, Dahai Zhang, Haijun Teng, Haijiang Lu

**Affiliations:** 1 Department of Orthopedic Surgery, Weifang Traditional Chinese Medicine Hospital, No. 1055 Weizhou Road, Kuiwen District, Weifang, Shandong China; 2 Department of Medical Affairs, The Second Naval Hospital of Southern Theater Command of PLA, No. 86 Sanya Bay Road, Tianya District, Sanya, Hainan China; 3 Department of Orthopedic Surgery, Affiliated Hospital of Weifang Medical University, No. 2428 Yuhe Road, Kuiwen District, Weifang, Shandong China; 4 Department of Orthopedic Surgery, The 80th Army Hospital of PLA, No. 256 Beigongxi Street, Weicheng District, Weifang, Shandong China

**Keywords:** Odontoid fracture, Anterior, Screw fixation, Percutaneous, Bony union

## Abstract

**Background:**

Percutaneous anterior odontoid screw fixation for odontoid fractures remains challenging due to the complex anatomy of the craniocervical junction. We designed a new guide instrument to help with the placement of guide wire, which have achieved satisfying surgical results. The objective of this study is to evaluate the safety and efficacy of this new tool in percutaneous anterior odontoid screw fixation.

**Methods:**

Twenty-nine patients with odontoid fracture were retrospectively evaluated. All patients underwent percutaneous anterior odontoid screw fixation with the traditional guide instrument (*n* = 13) or the new guide instrument we designed (*n* = 16). The following clinical outcomes were compared between the two groups: operation time, radiograph times, incision length, blood loss, postoperative hospitalization, postoperative complications, bony union, fixation failure, and reoperation. Radiographs or CT scans were performed at 3, 6 and 12 months after surgery.

**Results:**

There were no significant differences in preoperative demographic data between the two groups. The operation time (56.62 ± 8.32 Vs 49.63 ± 7.47, *P* = 0.025) and radiograph times (26.54 ± 6.94 Vs 20.50 ± 5.02, *P* = 0.011) of the designed guide instrument group were significantly lower than those of the traditional guide instrument group. There were no significant differences in incision length (16.08 ± 3.07 Vs 15.69 ± 2.73, *P* = 0.720), blood loss (16.08 ± 4.96 Vs 17.88 ± 5.98, *P* = 0.393), postoperative hospitalization (7.15 ± 1.91 Vs 6.88 ± 2.36, *P* = 0.734), postoperative complications (7.7% Vs 12.5%, *P* = 1), and bony union (92.3% Vs 93.8%, *P* = 1) between the two groups. No fixation failure or reoperation occurred in either group.

**Conclusions:**

The top of our designed guide instrument is a wedge-shaped tip with 30° inclination, which has a large contact area with the anterior surface of the cervical vertebra. According to our retrospective study, the guide instrument can reduce the operation time and radiograph times. It has potential clinical value, which needs further testing with a higher level of research design.

**Supplementary Information:**

The online version contains supplementary material available at 10.1186/s12891-020-03929-4.

## Introduction

Odontoid fractures are common, which represent approximately 9 to 20% of all cervical spine fractures [1–4]. Its treatment remains challenging due to the complex anatomy of the craniocervical junction [5]. It is generally accepted that type I and type III odontoid fractures based on the classification of Anderson and D’Alonzo can be treated by conservative strategies such as cervical orthoses, halo vests, and rigid cervical collars [5–7]. Type II fractures are mechanically unstable injuries [5, 8]. Conservative treatment for this type is associated with high bony nonunion rate, accordingly, surgical treatment is recommended [1, 5]. Over the past few decades, the applications of several surgical strategies (odontoid screw fixation, Magerl technique, and Harms technique) have achieved satisfying clinical results [3, 9–13]. It was reported that posterior approaches can achieve high bone union rate [14, 15], but they inevitably sacrificed atlantoaxial rotational motion [5, 15–17]. Anterior odontoid screw fixation can preserve normal atlantoaxial rotation [5, 8, 15, 16, 18] with union rate comparable to posterior approaches [17]. It is considered as the preferred treatment for odontoid fractures [17]. Percutaneous odontoid screw fixation is minimally invasive, which can shorten operation time and reduce blood loss compared with open technique [8].

The odontoid screw is percutaneously placed with the help of the guide tube, guide wire, and protection tube, which requires an experienced surgeon. The precise insertion of the guide wire into the ideal position is crucial and challenging. We have developed a new guide instrument to help with the placement of guide wire, which have achieved satisfying surgical results. In this report, the authors discuss the clinical application of this new tool.

## Methods

### Patient population

The clinical data of 37 patients with fresh type II odontoid fracture who underwent odontoid screw fixation in our hospital before June 2019 were retrospectively analyzed. The inclusion criteria were: the time from trauma to surgery was less than 30 days; the fracture was non-pathological; the surgical procedure was percutaneous; and the follow-up time after surgery was at least 6 months. Our contraindications for odontoid screw fixation included comminuted fracture, severe osteoporosis, severe cervicothoracic kyphosis, transverse ligament rupture, fracture line from anterior inferior to posterior superior of odontoid base, and non-reducible fractures. Excluding seven patients who underwent open screw fixation and one patient who had insufficient follow-up period, 29 patients met the inclusion criteria, whose mean age was 48.6 years (range, 31 to 78 years). We treated 13 of the patients with the traditional odontoid screw guide instrument before December 2013, and 16 of the patients with the new guide instrument we designed after December 2013. The study had been approved by the ethics committee of our hospital.

### Description of the new guide instrument

**Fig. 1:**
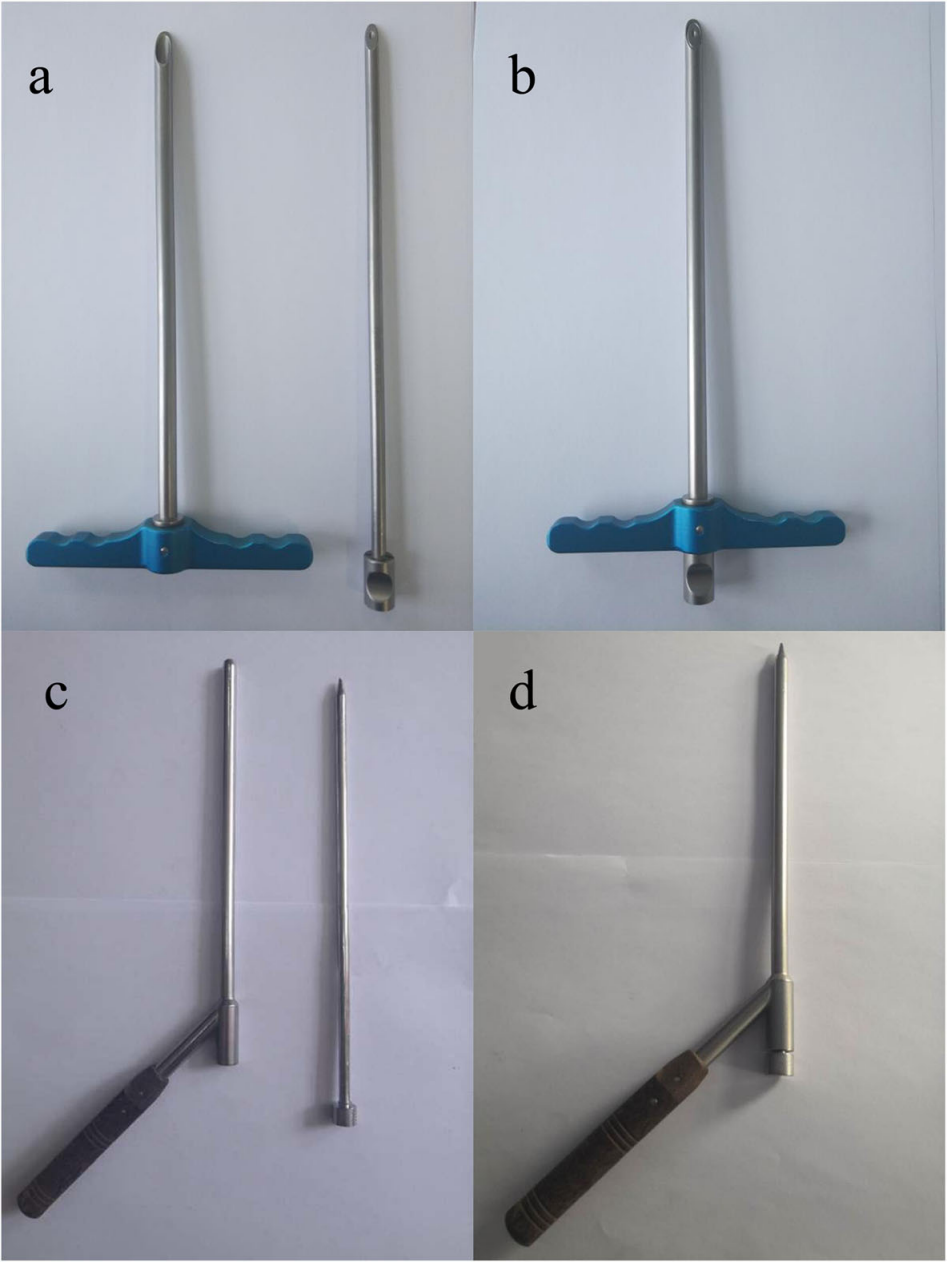
Guide instruments. **a**, **b** Designed guide instrument; **c**, **d** Traditional guide instrument

One of the authors (HT) developed a new system of guide instrument (Chinese patent number: 2017 21,295,183.4, Shanghai Sanyou Medical Equipment Co., Ltd., Shanghai, China, Fig. 1a and b) to facilitate the placement of odontoid screw percutaneously, which included the following components:

A guide tube which is 1.3 mm in internal diameter, 6.0 mm in external diameter, and 250 mm in length with a wedge-shaped tip of 30° inclination.

A protection tube which is 6.1 mm in internal diameter, 8 mm in external diameter, and 235 mm in length with a wedge-shaped tip of 30° inclination.

The traditional guide instrument (Medtronic Sofamor Danek USA Inc., Memphis, TN) has a blunt tip structure, which is shown in Fig. 1c and d.

### Radiological assessment

All fractures were assessed preoperatively by the lateral and open-mouth anteroposterior radiographs and computed tomography (CT) scans with reconstructions. The severity of spinal cord injury was evaluated by magnetic resonance imaging (MRI). Radiographs were performed at 3, 6 and 12 months postoperatively and annually thereafter. Bony union was defined as evident bridging bone across the fracture line on the radiographic images. If nonunion was suspected, CT scans and reconstruction were performed to confirm its occurrence.

### Surgical technique

#### Preoperative preparation

Gardner-Wells skull traction with a weight of 1-3 kg was performed preoperatively for all patients with odontoid fractures to reduce and stabilize the fracture. Anatomic or near-anatomic reduction of the fracture must be ensured before surgery. We simulated entry points and screw directions on radiography and CT scans and determined the optimal position of the screw. General anesthesia was carefully carried out with skull traction to avoid fracture dislocation and secondary risks caused by cervical hyperextension. The patients were positioned supine on the operating table with a cushion behind the shoulders to slightly extend the neck. A roll of gauze was placed in the patient’s mouth to maintain an open mouth for intraoperative radiography.

#### Surgical procedure

**Fig. 2:**
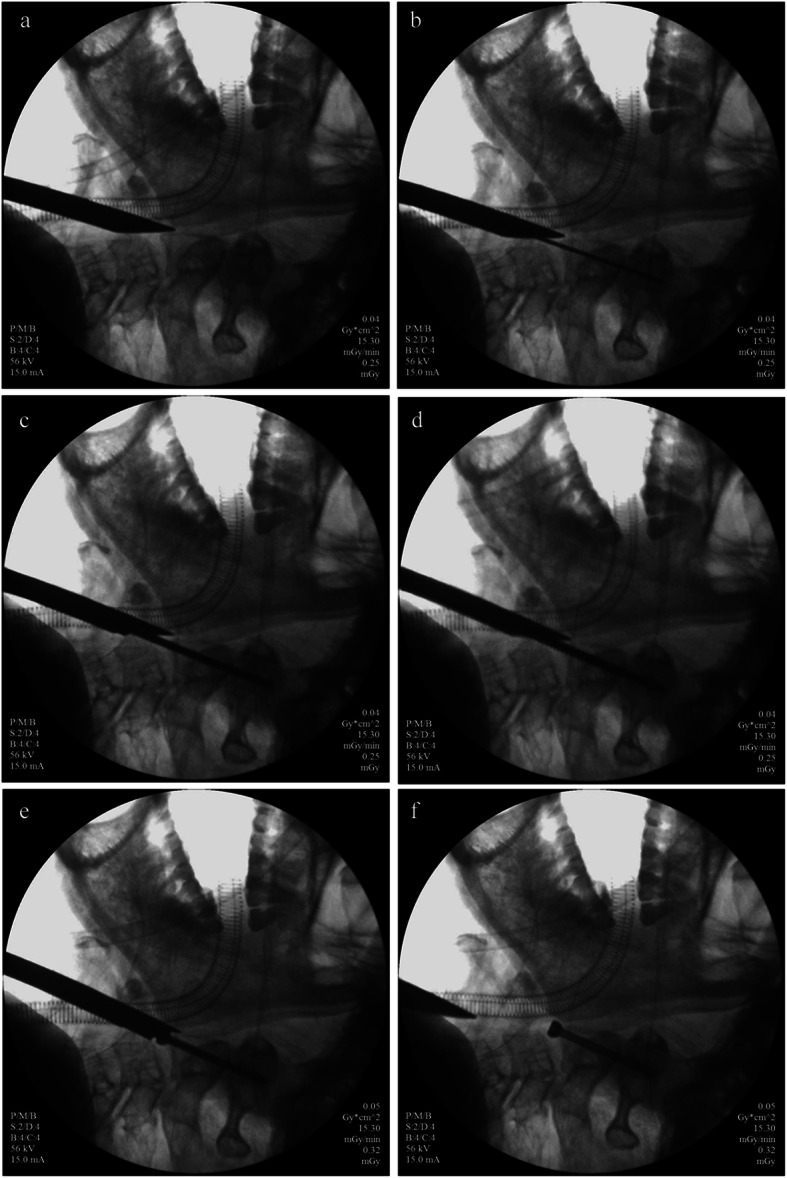
Work steps of our guide instrument. **a** The guide instrument (guide tube and protection tube) was placed at the desired entry point; **b** The guide wire was advanced to the posterior superior tip of the odontoid across the fracture line; **c** The drill bit was advanced to the posterior superior tip of the odontoid along the guide wire; **d** The tap was advanced along the guide wire to enlarge the trajectory; **e** the proper cannulated screw was advanced to the posterior superior tip of the odontoid along the guide wire and the apical cortical bone of the odontoid was penetrated; **f** The guide wire and the protection tube were removed

**Fig. 3:**
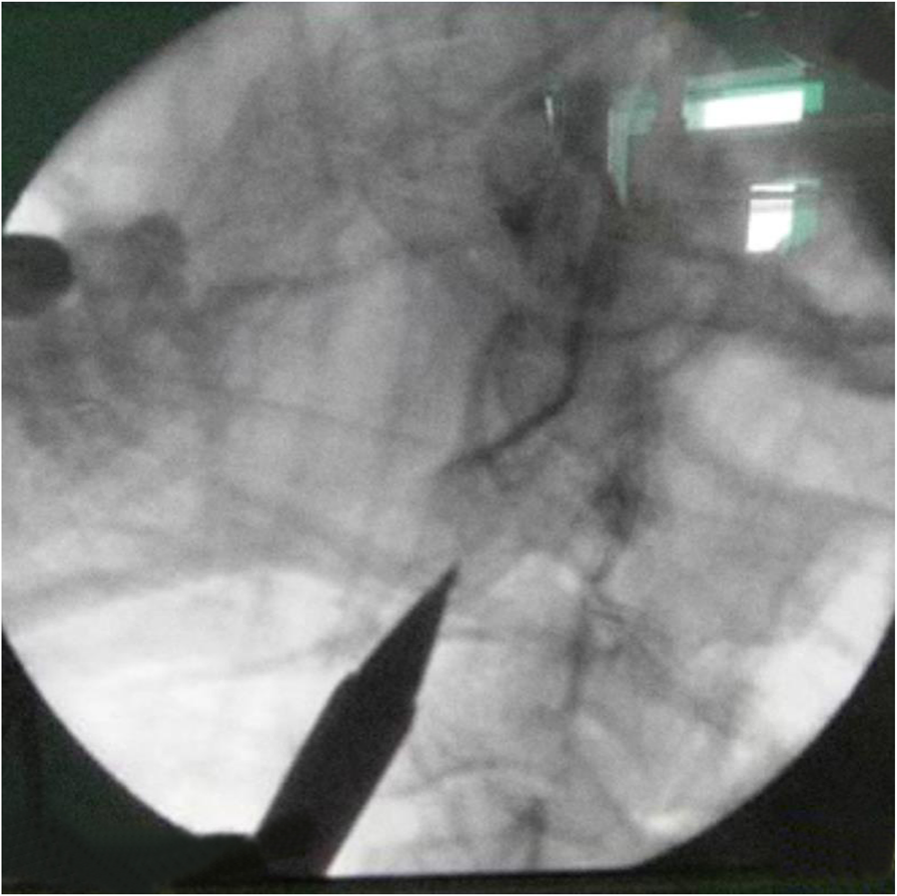
The traditional guide instrument (guide tube and protection tube) was placed at the desired entry point. This blunt tip structure slid easily when positioning

**Fig. 4:**
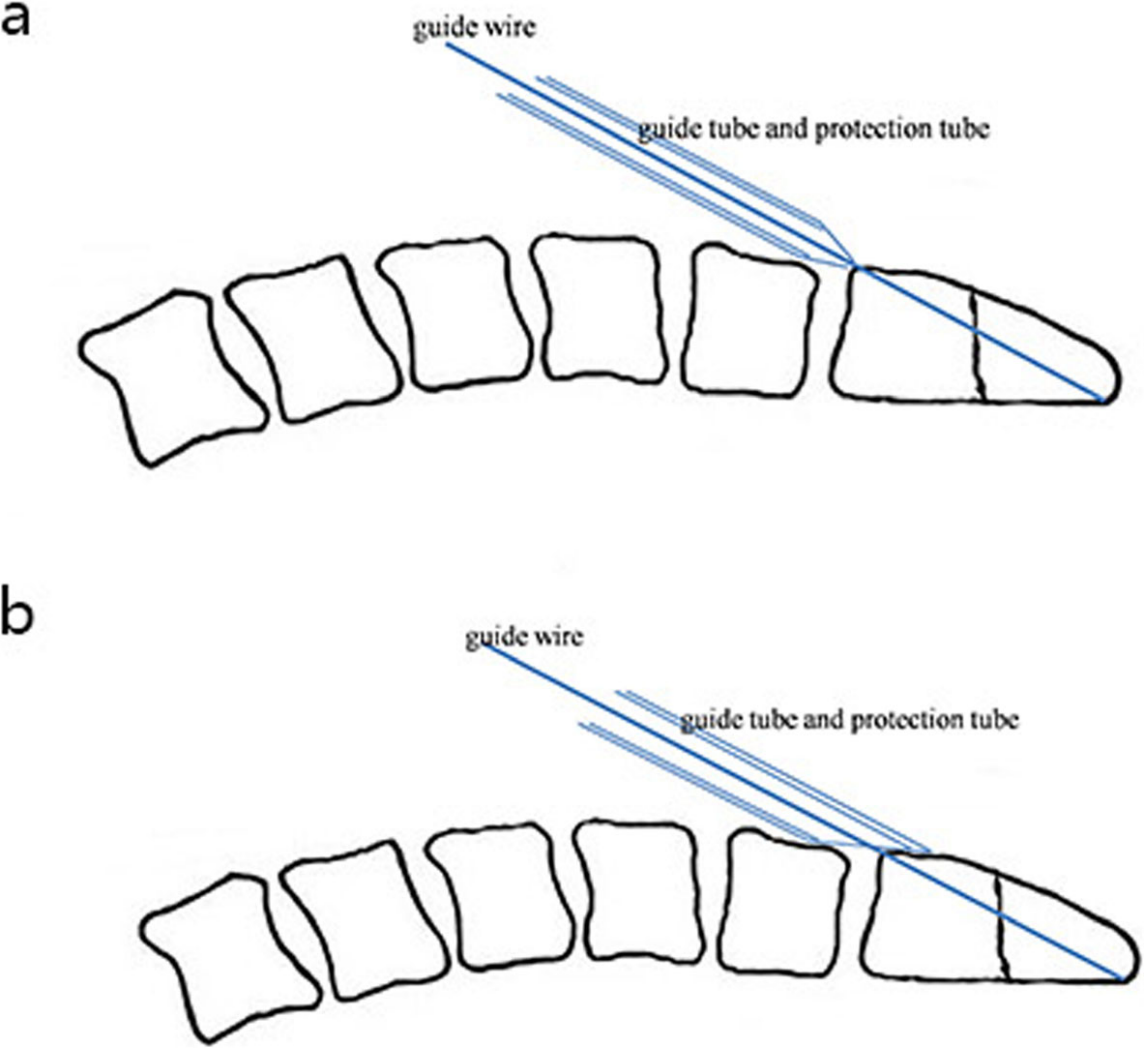
Illustrative diagram of the two guide instruments. **a** The traditional guide instrument; **b** The designed guide instrument

A 1–2 cm incision was performed along the medial edge of the right sternocleidomastoid muscle at approximately the C4–C5 level. The platysma and the fascia of the sternocleidomastoid were bluntly divided by a hemostat. Blunt dissection was performed along the potential space between the carotid sheath and trachea-oesophageal complex with the aid of the guide instrument, until the anterior surface of the vertebra was reached. Then the guide instrument cephalad extended to the middle C2/3 disc space and the anteroinferior area of C2 (Fig. 2a). Keeping the end face of the guide instrument close to the anterior surface of the cervical vertebra, we inserted the guide wire into the guide tube, slightly adjusted the position and direction of the guide instrument so that the guide wire passed through the fracture line from the anteroinferior lip of the C2 to the posterior superior tip of the odontoid with the help of the power-drill (Fig. 2b). In order to get the optimal trajectory, the insertion of guide wire often required more than one time. This step was crucial because the position of the guide wire in the odontoid was the position of the screw. The guide tube was then removed. The penetration depth of the guide wire was measured by the depth gauge. With the help of the power-drill, the drill bit reached the posterior superior tip of the odontoid along the guide wire (Fig. 2c). Care must be taken to prevent the guide wire from advancing and damaging the spinal cord. Then the drill bit was removed. The tap was advanced along the guide wire to enlarge the trajectory (Fig. 2d). Finally, the proper cannulated screw was advanced through the tapped hole with the help of the cannulated hexagon screwdriver. The apical cortical bone of the odontoid needed to be penetrated. As the screw was tightened, the distal fracture fragment was pulled toward to the proximal fragment and the fracture line was compressed (Fig. 2e). The guide wire and the protection tube were removed (Fig. 2f). The position and stability of the cannulated screw were checked, and the incision was checked for bleeding. The above steps were completed under continuous radiograph monitoring. A single suture closed the incision. Figure 3 showed the positioning process of the traditional guide instrument. Figure 4 illustrated the structural difference between the two guide instruments. Our designed guide instrument with a wedge-shaped tip has a large contact area with the anterior surface of the cervical vertebra, which makes it harder to slip during operations compared with the traditional ones. For all the 29 patients, the fracture was fixed with a single screw. Two experienced spinal surgeons performed the surgical procedures.

#### Postoperative management

Radiograph was performed on the postoperative day-one. All patients were allowed to walk on the postoperative day-two, and they were immobilized in a soft cervical collar postoperatively for approximately 12 weeks.

### Data collection

Demographic data (age, cause of injury, fracture types, associated spinal cord injury, associated C1 fracture, time between trauma and operation, and follow-up duration) and clinical outcomes (operation time, radiograph times, incision length, blood loss, postoperative hospitalization, postoperative complications, bony union, fixation failure, and reoperation) were collected for all patients in both groups. Fracture types were based on the Grauer classification system [7]. Associated spinal cord injury was assessed by American Spinal Injury Association (ASIA) standards. Fixation failure referred to screw breakage or screw loosening and exit. Postoperative complications included hematoma, neurologic deterioration, swallowing dysfunction, vocal cord dysfunction and respiratory dysfunction.

### Statistical analysis

The continuous variables were represented as mean ± standard deviation. Because of the small sample size, the two groups were compared using the Student’s t-test or Wilcoxon rank sum test for continuous variables, and the Fisher’s exact test for categorical variables. Statistical analysis was performed using SPSS 21 software (SPSS Inc., Chicago, IL, USA). *P* values less than 0.05 were considered to be statistically significant.

## Results

### Comparison of preoperative demographic data

The mean follow-up duration was 16.1 months with a range of 6 to 48 months. There were no significant differences in age, cause of injury, fracture type, associated spinal cord injury, associated C1 fracture, and time between trauma and operation between the two groups (*P* > 0.05). Table 1 showed the preoperative demographic data in the traditional guide instrument group and the designed guide instrument group.

**Table 1:** Comparison of preoperative demographic data between traditional guide instrument group and designed guide instrument group

Characteristics	Statistics		*P* value
	Traditional guide instrument	Designed guide instrument	
Patients (*n*)	13	16	
Age (years)	49.31 ± 10.90	47.94 ± 11.89	0.751
Cause of injury (*n*)			0.573
Traffic accidents	5	4	
Fall from height	6	7	
Falls from standing	2	5	
Fracture type (*n*)			0.715
Type II A	7	7	
Type II B	6	9	
Type II C	0	0	
Associated spinal cord injury (n)			1.000
ASIA Grade C	0	1	
ASIA Grade D	3	3	
Associated C1 fracture (n)	3	4	1.000
Time between trauma and operation (d)	3.77 ± 1.74	3.50 ± 1.37	0.644
Follow-up duration (months)	15.38 ± 9.67	16.75 ± 10.28	0.718

### Comparison of clinical outcomes

**Fig. 5:**
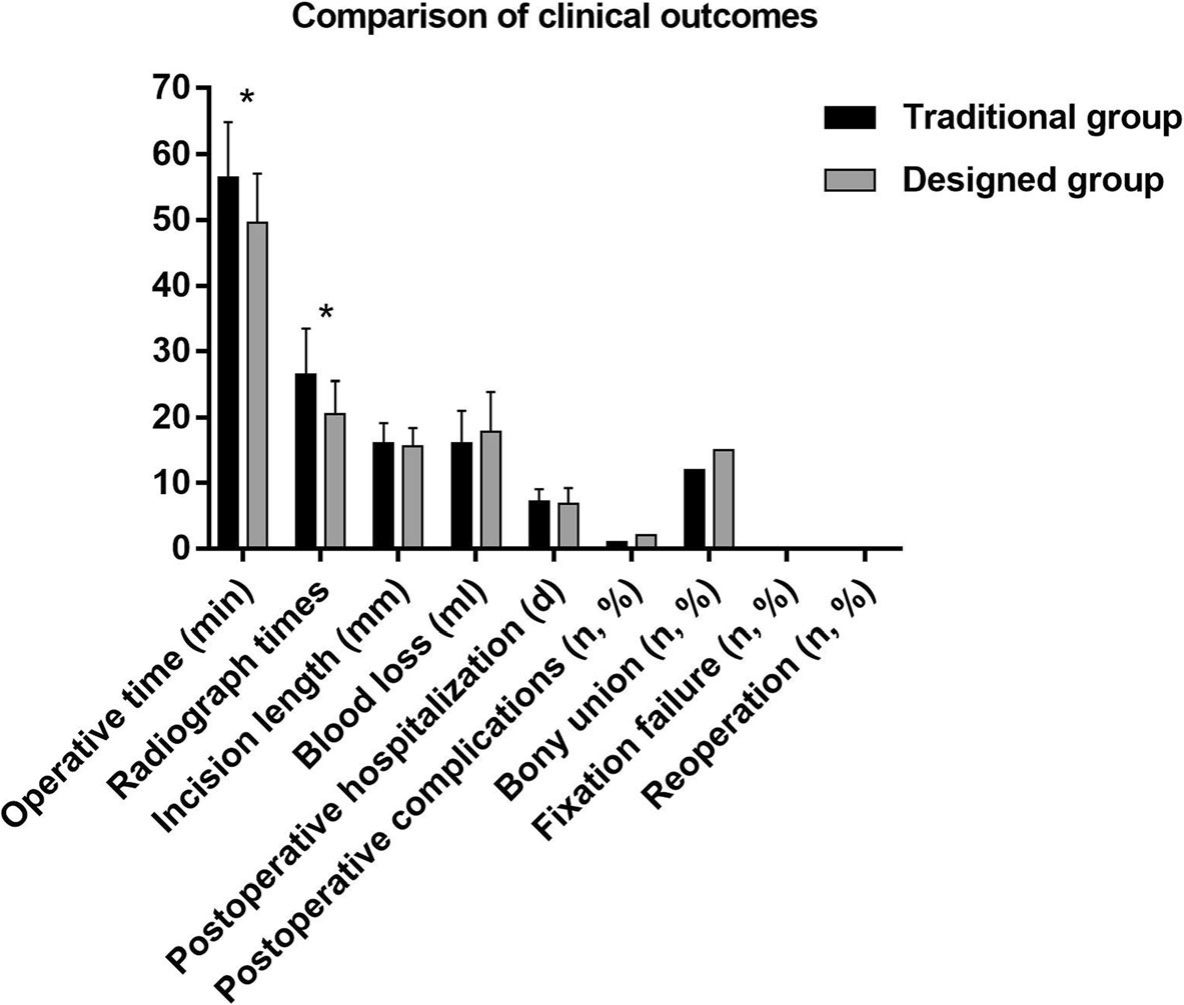
Comparisons of clinical outcomes between traditional guide instrument group and designed guide instrument group. (*: *P* < 0.05)

**Fig. 6:**
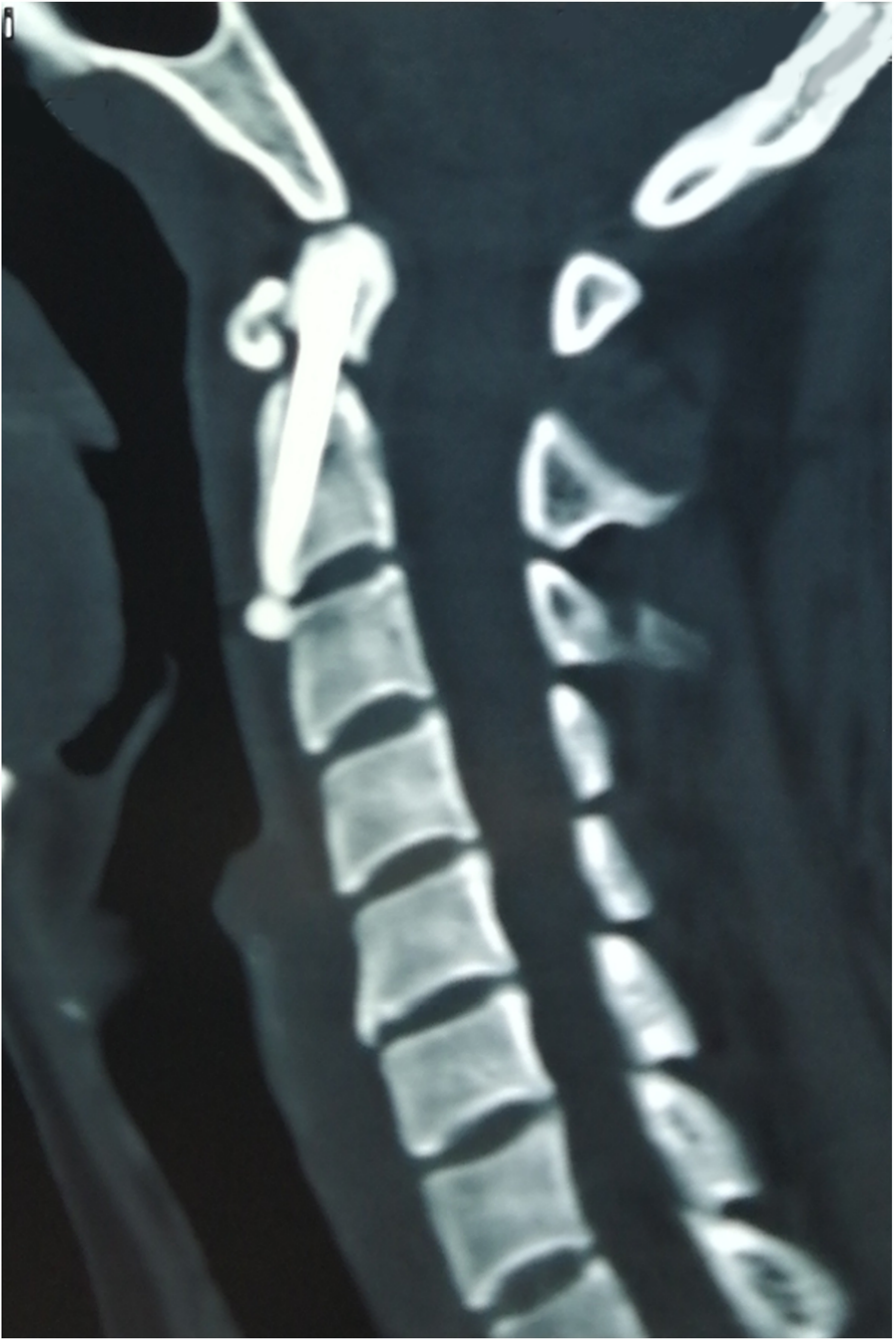
The CT scans showed screw fixation and nonunion of the odontoid fracture 6 months after surgery. Clear fracture line was found with osteosclerosis

**Fig. 7:**
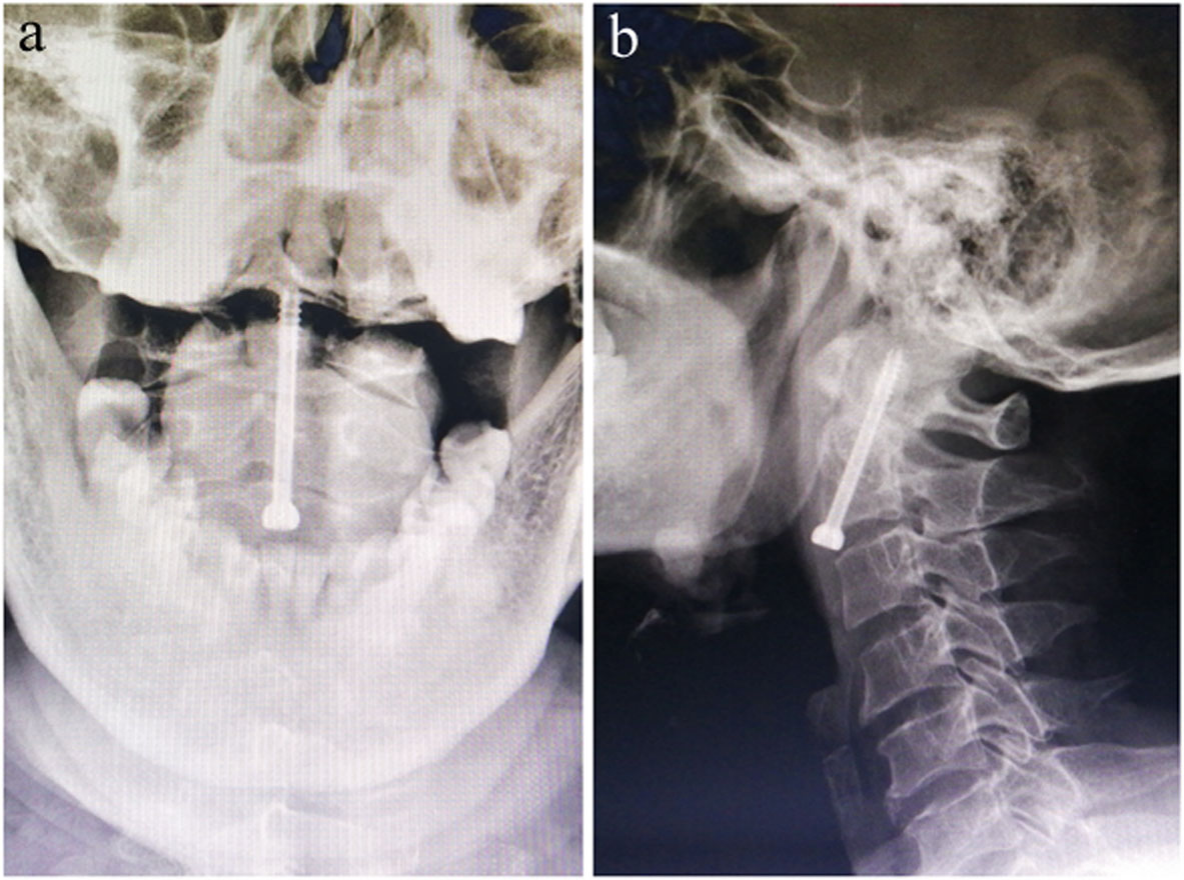
The anteroposterior (**a**) and lateral (**b**) radiograph showed screw fixation and union of the odontoid fracture 3 months after surgery. The fracture line was unclear with bridging bone passing

Compared with the traditional guide instrument group, the operation time (56.62 ± 8.32 Vs 49.63 ± 7.47, *P* = 0.025) and radiograph times (26.54 ± 6.94 Vs 20.50 ± 5.02, *P* = 0.011) of the designed guide instrument group were significantly lower. There were no significant differences in incision length (16.08 ± 3.07 Vs 15.69 ± 2.73, *P* = 0.720), blood loss (16.08 ± 4.96 Vs 17.88 ± 5.98, *P* = 0.393), postoperative hospitalization (7.15 ± 1.91 Vs 6.88 ± 2.36, *P* = 0.734), postoperative complications (7.7% Vs 12.5%, *P* = 1), and bony union (92.3% Vs 93.8%, *P* = 1) between the two groups. No fixation failure or reoperation occurred in either group. All clinical data are displayed in Fig. 5. The neurological function of 7 patients with spinal cord injury recovered within 3 months after surgery. No hematoma, neurologic deterioration, and respiratory dysfunction occurred in either group. One case of swallowing dysfunction and one case of vocal cord dysfunction occurred in the designed guide instrument group. Additionally, one case of swallowing dysfunction occurred in the traditional guide instrument group. These complications faded gradually and spontaneously without therapy. No fixation failure occurred in either group. One case of nonunion was observed in each group. The last two follow-up dates were 6 months and 1 year after surgery, respectively (Fig. 6). Both patients with nonunion refused posterior C1–C2 fixation and fusion due to the absence of complaints or instability. Bony union was found for the other 29 patients between 3 to 6 months after surgery (Fig. 7).

## Discussion

Fractures at the odontoid base, classified as Type II injuries by Anderson and D’Alonzo system, are the most common type (more than 60%) of all odontoid fractures [8, 11, 14, 15]. They were considered relatively unstable and had a high incidence of nonunion [5]. Anterior odontoid screw fixation can achieve a high union rate (86–100%) as found in numerous studies [5, 8, 15, 19–23]. It has a number of advantages, including immediate stability, less postoperative pain, and direct fracture fixation without bone graft [16]. More importantly, normal physiologic atlantoaxial rotation can be preserved [5, 8, 16]. However, the application of anterior odontoid screw fixation has three prerequisites: suitable anatomy of the odontoid without atlantoaxial dislocation, suitable fracture morphology, and suitable bone quality without osteoporosis [9]. Contraindications of anterior odontoid screw fixation include comminuted fracture, severe osteoporosis, severe cervicothoracic kyphosis, late fractures, ligament transverse rupture, posterior oblique fracture line, non-reducible fractures, and nonunion for more than 3 months [4, 14, 16, 17, 24–26]. Posterior cervical spine fixation should be performed in patients with these contraindications [14, 15, 17].

In our case series, all patients had no contraindications of anterior odontoid screw fixation. The age, cause of injury, fracture type, associated injury, and time from trauma to operation showed a similar pattern in the traditional guide instrument group and the designed guide instrument groups. Grauer et al. [7] further classified type II fractures into IIA, IIB and IIC fractures, representing nondisplaced fractures, anterior superior to posterior inferior or displaced transverse fractures, and anterior inferior to posterior superior or comminuted fractures based on the fracture line obliquity, displacement and comminution to guide treatment, respectively. In our case series, type IIA and IIB odontoid fractures were treated with anterior odontoid screw fixation.

Open technique for odontoid screw fixation requires intraoperative extensive exposure and placement of drainage postoperatively [5]. Kazan et al. [27] developed a telescopic tube system and demonstrated the feasibility of percutaneous odontoid screw fixation technique on the cadavers, which was the first report of percutaneous odontoid screw fixation. Similarly, Horgan et al. [28] developed an endoscopic approach with a soft tissue dilator. Wu et al. [29] developed a two-hole guide tube to facilitate the making of a second optimal kirschner wire trajectory when an initial suboptimal kirschner wire hole was drilled. However, in our opinion, the distance between the two holes was fixed, and the position of the first Kirschner wire would affect the position of the second Kirschner wire. Chi et al. [5] developed a system of tools with a blunt tip-guide tube and achieved satisfying results in the clinical application of 10 patients. Wang et al. [8] compared percutaneous and open anterior screw fixation for odontoid fractures prospectively and found that the former can significantly shorten operation time, reduce surgical exposure and blood loss. Umana et al. [4] used a ruler in the X-ray monitor to evaluate the final trajectory of the kirschner wire and to make the adjustments needed. They also developed a soft tissue dilator to protect the soft tissue using an endotracheal tube with an internal diameter of 6 mm. However, without a guide instrument, there was no positioning and fixing function of the guide instrument to the kirschner wire. The guide instruments used by the above three authors [5, 8, 29] have a similar structure - a guide tube with a blunt tip. The guide tube we used before also had a blunt tip structure. We considered this guide tube with a blunt tip was prone to sliding on the anterior surface of the vertebra, especially when the power-drill was working. Compared to these guide instruments, the top of our designed guide instrument (guide tube and protection tube) has a wedge-shaped tip with 30° inclination, which has a larger contact area with the anterior surface of the cervical vertebra. It is more stable when positioning. It is helpful to guide the guide wire into the odontoid accurately and quickly. Moreover, it can cover the tip of the guide wire to prevent it from stabbing important tissues such as the esophagus and blood vessels. Compared with the traditional guide instrument with a blunt tip, our guide instrument significantly decreased operation time and radiograph times. The short operation time reflects the effectiveness of our new guide instrument. The less radiograph times reduce the injury to clinicians. Regardless of the magnitude of the difference, the reduction of these two parameters is of great significance for the clinic. However, this is a retrospective study. And the sample size is small. A higher level of research design such as large-sample-size randomized controlled trials should be conducted to further study.

Fracture reduction before anterior odontoid screw fixation is critical. Displacement of the fracture should be less than 3 mm in our series before surgery if the fractures were failed to anatomic reduction. After traction, most of our patients with fracture displacement met the surgical requirements. The length of the screw is also critical. Ideally, the screws should just break through the apical cortical bone of the odontoid to compress the fracture line when the screw is tightened. If the screw is too short, its pull on the odontoid will not be enough, which will easily cause reduction failure. If the screw is too long, it may damage the spinal cord or lose its compression.

In this study, we used a single screw for percutaneous procedure. Studies have shown that there were no significant differences in bony union rate [30] and biomechanical stability [31, 32] between a single screw and two screws fixation. In order to ensure sufficient screw trajectory and enough cortical bone in front of the screw, many surgeons described that the ideal entry point was mostly at the C2/3 disc level [8]. In our series, most screws entered through the anterior edge of the C2/3 disc. The impact of slight injury in the anterior edge of C2/3 disc on patients needs further study.

Due to the complex anatomy of the craniocervical junction and the high precision requirements of odontoid screw fixation, percutaneous anterior odontoid screw fixation may be technically challenging, especially for novice surgeons without minimally invasive spinal surgery experience [8]. Our guide instrument reduces the difficulty and learning curve of the surgery to a certain extent because it facilitates the placement of screw. However, this study has certain limitations. Firstly, the time span of the study is long. The proficiency of the surgeon affects the surgical outcomes. In addition, the follow-up time varies, some patients lack long-term follow-up results. Thirdly, there are only 13 samples in the traditional group and 16 samples in the designed group. The small sample size may affect the results. It is also a single-center, retrospective study. In view of these limitations, prospectively planned, multicenter and larger-sample trials in the future are essential for clinicians to treat and manage patients.

## Conclusions

According to this retrospective study, our guide instrument significantly decreased operative time and radiograph times compared with the traditional guide instrument with a blunt tip. It has potential clinical value, which needs further testing with a higher level of research design such as large-sample-size randomized controlled trials.

## Supplementary Information

**Additional file 1.**

## Data Availability

The raw data used and/or analyzed during the current study are available from the corresponding author on reasonable request.

## Abbreviations

CT: Computed tomography
MRI: Magnetic resonance imaging
ASIA: American Spinal Injury Association
